# Cardiovascular co-morbidity in cancer patients: the role of psychological distress

**DOI:** 10.1186/s40959-016-0019-x

**Published:** 2016-11-15

**Authors:** Dounya Schoormans, Susanne S. Pedersen, Susanne Dalton, Nina Rottmann, Lonneke van de Poll-Franse

**Affiliations:** 1 Department of Medical and Clinical Psychology, Center of Research on Psychology in Somatic diseases (CoRPS) Tilburg University, Warandelaan 2, Tilburg, 5000 LE The Netherlands; 2 Department of Psychology, University of Southern Denmark, Odense, Denmark; 3 Department of Cardiology, Odense University Hospital, Odense, Denmark; 4 Survivorship Unit, Danish Cancer Society Research Center, Copenhagen, Denmark; 5 Department of Public Health, National Research Center for Cancer Rehabilitation, Research Unit of General Practice, University of Southern Denmark, Odense, Denmark; 6 Division of Psychosocial Research and Epidemiology, Netherlands Cancer Institute, Amsterdam, The Netherlands; 7 Comprehensive Cancer Organization Netherlands, Eindhoven, The Netherlands

**Keywords:** Cancer, Survivors, Cardiovascular, Psychological distress, Mechanisms

## Abstract

Due to aging of the population and cardiotoxic cancer treatment, there is an increasing group of patients with cancer and co-morbid cardiovascular disease (CVD). In order to find a balance between the risk of undertreating the malignancy on the one hand and inducing CVD on the other hand, CVD risk stratification at the time of cancer diagnosis and knowledge on the pathway for developing incident CVD in cancer patients is vital. In this paper, we propose an adapted multiple-hit hypothesis for developing CVD in cancer patients describing that patients with cancer are exposed to a series of sequential or concurrent events that together make them more vulnerable to reduced cardiovascular reserves, development of incident CVD and ultimately death. We highlight the possible impact of psychological distress secondary to a cancer diagnosis and/or treatment, which in turn may increase the risk of incident CVD in patients diagnosed with cancer. Furthermore, we discuss potential behavioral and pathophysiological mechanisms underlying the link between psychological distress and the pathophysiology of incident CVD. In addition, key unanswered questions for future research are posed. In the future, researching the adapted multiple-hit hypothesis for developing CVD among cancer patients will hopefully advance the care of cancer patients by finding some of the missing pieces of the puzzle. To do so, we need to focus on minimizing cardiovascular risk and promoting cardiovascular health in cancer patients by addressing the knowledge gaps formulated in this paper.

## Cardiovascular co-morbidity in cancer patients

Due to aging of the population, there is an increasing number of patients with cancer and co-morbid cardiovascular disease (CVD). Additionally, cancer patients may develop incident CVD as a result of the cardiotoxicity of cancer treatment. Fortunately, there has been increasing awareness of co-morbid CVD in cancer patients. In 2009, the International CardiOncology Society (ICOS) was established [1]. ICOS advocates a multi-disciplinary effort to combat CVD in cancer patients by both training oncologists and cardiologists and conducting multidisciplinary research. In 2014, during the international colloquium on cardio-oncology, ICOS stressed the importance of finding a balance between the risk of undertreating the malignancy on the one hand and inducing CVD by overtreating on the other hand [2]. In order to accomplish this goal, they emphasize the importance of CVD risk stratification at the time of cancer diagnosis. Recommendations based on consensus have also been published to target the vulnerable cancer patient population, emphasizing the importance of a thorough cardiac evaluation, including the assessment of risk factors, prior to formulating a cancer treatment plan [3].

## Adapted multiple-hit hypothesis: the impact of psychological distress

**Fig. 1:**
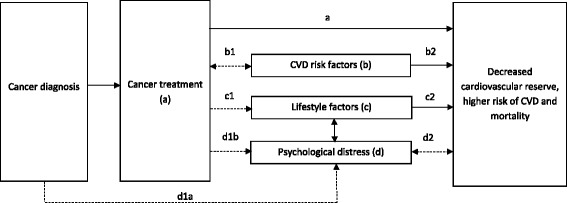
The adapted multiple-hit hypothesis on the pathogenesis of decreased cardiovascular reserve, higher CVD risk, and mortality among cancer patients. Note: Please note that the current model is a necessary simplification as various factors are associated with one another. To maintain the comprehensibility of the model, not all associations are explicitly depicted in the model. Interrupted arrows indicate relationships added to the original model, whereas the uninterrupted arrows describe the original relations or highlight the increasingly acknowledged associations. Please note, that both bidirectional and unidirectional (causal) arrows are depicted

Important in CVD risk stratification is knowledge of the pathway for developing CVD in cancer patients. Within the field of cardio-oncology there is increasing interest in the multiple-hit hypothesis for developing CVD in cancer patients. This hypothesis was first described by Jones et al. in 2007 [4], and proposes that patients with cancer are exposed to a series of sequential or concurrent events that together make them more vulnerable to reduced cardiovascular reserves, development of incident CVD and ultimately death. With new developments in the field of cardio-oncology and by including knowledge on psychological distress as a risk factor for the pathogenesis of CVD in non-cancer populations, we have adapted the multiple-hit hypothesis (Fig. 1). In this paper, we will describe the adapted hypothesis in more detail, focusing on psychological distress secondary to a cancer diagnosis and/or treatment, which in turn may increase the risk of incident CVD in patients diagnosed with cancer. In the model, depicted arrows represent the most dominant associations, although many other relations exist. It should be noted that the current model depicted in Fig. 1 is a necessary simplification as various factors are associated with one another. To maintain comprehensibility of the model, not all associations will be explicitly depicted in the model, yet many of these associations are addressed throughout the paper.

### Cancer treatment (a)

Cancer treatment consists of radiation, chemo-, hormone, targeted-, or immunotherapy or a combination of those. Some of these cancer treatments are cardiotoxic. Chemotherapy with anthracyclines as well as radiation to the chest in treating lymphomas or breast cancer are two examples of cardiotoxic cancer treatments [5, 6]. These cardiotoxic treatments can lead to reduced cardiovascular reserves and ultimately a heterogeneous group of CVDs ranging from benign to potentially lethal, represented by the unidirectional arrow ‘a’ (Fig. 1) [7, 8]. CVDs that have been associated with cancer treatments include thrombosis, electrocardiographic changes, arrhythmias, myopericarditis, myocardial infarction, cardiomyopathy, valvular heart disease, and congestive heart failure [5, 6]. These CVDs can occur within days, weeks, months or years after treatment [5]. Some CVDs are relatively treatment-specific. For example, heart failure is common as a sequel to treatment with anthracyclines [6], whereas thoracic radiation to the left side of the chest involving the heart has been associated with risk of valvular stenosis, restrictive cardiomyopathy, pericarditis, and myocardial infarction post treatment [7].

### CVD risk factors (b)

We view the relation between cancer treatment and CVD risk factors as bidirectional (arrow b1), with CVD risk factors leading to decreased cardiovascular reserves, CVD and mortality (unidirectional arrow b2). Having one or more CVD risk factors, such as older age and morbidities like hypertension or diabetes mellitus [9] prior to cancer diagnosis, affect cancer treatment options, often resulting in the use of less aggressive cancer treatment [10]. We believe that in light of the multiple-hit hypothesis, the presence of CVD risk factors may also be viewed as secondary to cancer treatment: Cancer treatments can induce CVD risk factors such as hypertension [11] and diabetes mellitus [12], decreasing cancer patients’ cardiovascular reserve and enhancing their risk of incident CVD and mortality. Additionally the effect of CVD risk factors may be enhanced or diminished by lifestyle factors such as impaired or increased physical activity or psychological distress like depressive symptoms (these associations are not depicted in the model). The direct effects of both lifestyle factors and psychological distress on the pathogenesis of CVD in cancer patients will be discussed below.

### Lifestyle factors (c)

Due to cancer and the intense nature of its treatment, patients may develop unhealthy lifestyles, including weight loss and physical inactivity, which may decrease their cardiac reserve and enhance their risk of CVD and mortality (depicted by the unidirectional arrows c1 and c2).

### Psychological distress (d)

Research on risk factors for the development and progression of CVD among non-cancer populations has shown that next to CVD risk factors like hypertension, the mere presence of psychological distress is bad for patients’ health outcomes. Symptoms of depression, anxiety, specific personality traits, and fatigue have all been shown to predict the onset of CVD and the prognosis in patients with established CVD, independent of traditional biomedical risk factors [13–21]. A recent meta-analysis based on 20 studies examining the predictive value of *anxiety* for the incidence of coronary heart disease in originally healthy individuals found that anxious people had a 26% increased risk of developing coronary heart disease and a 48% increased risk of cardiac death [14]. The so-called *Type D (‘distressed’) personality* has been consistently associated with poor outcomes in CVD-patients, as indicated in a recent meta-analysis [15]. Individuals with a Type D personality are characterized by a high score on both negative affectivity and social inhibition. Negative affect is the tendency to experience negative emotions, whereas social inhibition is the tendency to inhibit emotions and to experience a high level of insecurity in social encounters [22]. Also *fatigue* is predictive of new onset CVD in healthy individuals [16, 17] and coronary artery disease in CVD patients [18]. The psychosocial risk factor that has been studied the most is depression, with evidence pointing to a relation between *depression* and the development of new onset CVDs, such as heart failure [19, 20], increased risk of major adverse cardiac events [20, 21], and cardiac death in patients with established CVD [19].

It is well-known that suffering from cancer and additionally undergoing cancer treatment has a major impact on patients’ lives, with the majority of cancer patients experiencing psychological problems, such as feelings of anxiety, fatigue and depression [23, 24]. Moreover, one in 5 cancer patients have a Type D personality [25]. Hence, cancer patients may have an increased risk for onset CVD by these elevated levels of psychological distress after cancer diagnosis alone. It is important to note, however, that psychological distress could also be present *prior* to cancer diagnosis or throughout cancer treatment. Hence, some individuals may have already been at risk of developing CVD prior to being diagnosed or treated for cancer due to their prior experienced level of distress.

In 2007, when the original multiple-hit hypothesis was proposed, little was known about the potential putative role of psychological factors in CVD. Only in 2012, the European Society of Cardiology included psychosocial risk factors (e.g. depression and anxiety) in the Guidelines on Cardiovascular Disease Prevention in Clinical Practice for the first time [26]. In 2014, the American Heart Association followed with a Scientific Statement advocating that depression be given risk factor status at the same level as traditional risk factors, due to considerable evidence that depression is linked not only to CVD prognosis but also that depression is a risk factor for incident CVD [27]. More recent evidence implicating psychological distress as a risk factor for CVD and the fact that cancer patients experience high levels of psychological distress has inspired us to adapt the current multiple-hit hypothesis. In addition to including the causal effect of cancer diagnosis and treatment on experiencing psychological distress (unidirectional arrows d1a and d1b), we added the bidirectional relation between psychological distress and decreased cardiovascular reserve, CVD and mortality (bidirectional arrow d2), as psychological distress is not only a risk factor for incident CVD but is also increased in cancer patients who have co-morbid CVD.

### Behavioral and pathophysiological mechanisms involved in psychological distress as a risk factor

The literature on the role of psychological distress in the pathogenesis of CVD among non-cancer populations has suggested that various mechanisms - both of behavioral and pathophysiological nature - are involved. With respect to behavioral mechanisms, anxiety, Type D personality, and depression have been related to unhealthy lifestyles, such as smoking, poor treatment adherence and bad consultation behavior [28–34]. Alternatively, lifestyle factors such as increased physical activity have been associated with lower levels of psychological distress, such as symptoms of depression [35].

With respect to pathophysiological mechanisms, platelet dysfunction, autonomic nervous system dysregulation, hypothalamic-pituitary-adrenal-axis (HPA-axis) dysregulation, cellular ageing, and inflammatory activation have been suggested to be involved in the relation between psychological risk factors and CVDs. Platelet activation is increased in patients with depression [36]. Furthermore, thrombus formation on the surface of coronary plaque is key in acute coronary events, making platelet dysfunction a plausible mechanism [37]. In addition, patients with feelings of anxiety, depression, fatigue and a Type D personality have reduced heart rate variability (HRV) [38–41], which is an indicator of cardiac autonomic control and predictive of cardiovascular morbidity and mortality [42, 43]. Furthermore, the HPA-axis is known to play a role in CVD [44]. This system is vital for the regulation of the physiological reaction to stress, via the hormone cortisol, among others. Depressed, anxious, fatigued and Type D patients show elevated cortisol levels [44–47], suggesting that HPA-axis dysregulation is a possible mechanism. Likewise, anxiety and depression are related to shorter telomere length [48–50], a biomarker of cellular aging, which is in turn involved in the pathogenesis of CVD [51]. Finally, depression, anxiety, fatigue, and Type D personality have been associated with elevated levels of pro-inflammatory cytokines (e.g. interleukin-1 (IL), IL-6, TNF-alpha, and its soluble receptors sTNFR1 and sTNFR2) [52–55]. In turn, these cytokines are involved in the pathogenesis of CVDs, such as heart failure [56].

## Research agenda to study the adapted multiple-hit hypothesis

To date, there is limited research on the multiple-hit hypothesis for the development of CVD in cancer patients. Hence, there is an urgent need for research on the impact of psychological distress on incident CVD among cancer patients and the potentially additive or synergistic effects of cardiotoxic treatment, CVD risk factors, lifestyle factors and psychological distress, as described in the multiple-hit hypothesis. This knowledge will aid in risk stratification at the time of cancer diagnosis in order to (i) determine the appropriate cancer therapy and provide individually tailored treatment to decrease the risk of incident CVD, and (ii) identify patients at risk of incident CVD who could benefit from a disease management program to minimize the impact of CVD risk factors and psychological distress. For example, when treating a cancer patient with depression one would not only treat the depression but also reduce CVD risk and thus improve the patient’s quality of life and general health outcomes. Knowledge of the underlying behavioral and pathophysiological mechanisms is essential as it provides insight into understanding these relations, ultimately providing targets for intervention. In addition, information on clinical and patient-reported outcomes of cancer patients with co-morbid CVD will help tailor future care for this vulnerable patient group.

To answer these questions we can use various data sources, such as: (a) newly developed longitudinal in-depth data collections; (b) large population-based longitudinal aging studies; and (c) existing databases. Ideally, *newly developed longitudinal in-depth data collections* with long-term follow-up should be initiated, enabling a sufficient number of CVD events to occur. These studies should include information on all the aspects described in the multiple-hit hypothesis (i.e., type of cancer treatment, CVD risk factors, lifestyle factors, psychological distress, cardiovascular reserves, co-morbid CVD and mortality). Moreover, information on pathophysiological mechanisms, such as markers of inflammation or cellular aging, should be collected to gain insight into underlying mechanisms. By following oncology patients, one could examine the specific cardiotoxic effects and their interaction with CVD risk factors, lifestyle factors and psychological distress. Furthermore, *large population-based longitudinal aging studies* including originally healthy individuals would enable the identification of risk factors common to both cancer and CVD. It would also allow the examination of CVD risk factors present prior to a cancer diagnosis as healthy non-cancer patients would be included and followed over time until they first develop cancer and then pose the risk of developing CVD. These healthy individuals moreover offer matched cancer-free controls for each cancer patient as a reference group. *Existing databases* also offer unique opportunities to study parts of the research questions that can be extracted from our adapted multiple-hit hypothesis. For example, in the Netherlands, information from the southern region Netherlands Cancer Registry is linked to the PHARMO database network [57]. The population-based Netherlands Cancer Registry includes clinical information on type of malignancy, date of diagnosis and type of primary cancer treatment for all newly diagnosed cancer patients in the Netherlands. The PHARMO database is a network of electronic healthcare databases and combines data from primary and secondary healthcare settings in the Netherlands. Information on hospitalizations and drug dispensing records from the PHARMO database can be used to determine whether someone has co-morbid CVD, CVD risk factors (e.g. hypertension) or have a severe mental condition (e.g. clinical depression). These linked databases enable us to examine associations between cardiotoxic cancer treatment, CVD risk factors and psychological distress and their effect on the development of co-morbid CVD.

In conclusion, by researching the adapted multiple-hit hypothesis for developing CVD among cancer patients, we hope to find some of the missing pieces of the puzzle. Our ultimate goal is to advance the care of cancer patients. Therefore, we need to focus on the minimization of cardiovascular risk and promotion of cardiovascular health in cancer patients by addressing the knowledge gaps, as formulated above.

## Abbreviations

CVD: Cardiovascular disease
HPA: Hypothalamic-pituitary-adrenal-axis
HRV: Heart rate variability
ICOS: International CardiOncology Society
IL: Interleukin
